# Development of a framework for managing severe burns through a 17-year retrospective analysis of burn epidemiology and outcomes

**DOI:** 10.1038/s41598-021-88507-x

**Published:** 2021-04-30

**Authors:** Ling Chen, Xiaochong He, Jishu Xian, Jianmei Liao, Xuanji Chen, Yue Luo, Zonghua Wang, Ning Li

**Affiliations:** 1 Department of Emergency, The 958th Hospital of PLA, The Affiliated Hospital of Southwest Hospital, Third Military Medical University (Army Medical University), Chongqing, 400020 People’s Republic of China; 2 School of Nursing, Third Military Medical University (Army Medical University), Chongqing, 400038 People’s Republic of China; 3 Southwest Hospital, Third Military Medical University (Army Medical University), Chongqing, 400038 People’s Republic of China; 4 School of Public Health and Management, Chongqing Medical University, Chongqing, 400016 People’s Republic of China; 5 Institute of Burn Research, Southwest Hospital, Third Military Medical University (Army Medical University), Gaotanyan Street No.30, Shapingba District, Chongqing, 400038 People’s Republic of China

**Keywords:** Disease prevention, Trauma

## Abstract

Burns are one of the most common injuries in daily life for all ages of population. This study was to investigate the epidemiology and outcomes among burn patients in one of the largest burn centers in the southwest of China. The study was performed at the Institute of Burn Research in the first affiliated with the Army Medical University (AMU). A total of 17,939 burn patients were included in this retrospective study. Information regarding burn epidemiology and outcomes in 17 years were collected, calculated and compared. The age ranged from 257 days to 95 years old. Scalding and flame were the two most common causes to burn injuries, comprising of 91.96% in total. Limbs, head/face/neck, and trunk were the most frequently occurred burn sites, with the number and the percent of 12,324 (68.70%), 7989 (44.53%), and 7771 (43.32%), respectively. The average total body surface area (TBSA) was 13.64 ± 16.83% (median 8%) with a range of 0.1–100%. A total of 874 (4.9%) patients had TBSA > 50%. The presence of a burn with an inhalation injury was confirmed in 543 patients (3.03%). The average LOS was 32.11 ± 65.72 days (median: 17 days). Eventually, the retrospective analysis resulted in the development of a burn management continuum used for developing strategies to prevent and manage severe burns. The annual number of burn injuries has kept decreasing, which was partially attributed to the increased awareness and education of burn prevention and the improved burn-preventative circumstances. However, the burn severity and the economic burden were still in a high level. And the gender difference and age difference should be considered when making individualized interventions and rehabilitative treatments.

## Introduction

Burns are one of the most common injuries primarily resulted from heat, but can also be due to radiation, electricity, and chemicals^1^. Burns can be minor or life-threatening medical problems. In the European Union, burns are one of the three most common “unintentional fatal injuries”, together with poisoning and drowning (34.1%)^2^. According to the American Burn Association, it is reported that one in 1442 Americans died from injuries exposure to fire, flames, or smoke^3^.

In addition to enormous death of more than 250,000 annually, severe burn injuries can lead to unavoidable complications including scarring, disfigurement, physical disabilities, and require long-term rehabilitation, reconstruction and anti-scar therapy^4^. These physical problems are great challenges to maintain psychological health for patients and also cause major economic burden on family and society. Previous studies have revealed that burn patients experienced burn-associated abuse, stigma and rejection, resulting in high occurrence of mental health disorders including post-traumatic stress disorder (30%), anxiety (13–47%) and depression (23–61%)^5^. Moreover, burns are also among the most expensive traumatic injuries, because of long hospitalization and rehabilitation, and costly wound and scar treatment^2^.

The South-East Asian region including China is one of the three WHO regions with the greatest burden of injuries, accounting for almost half of the worldwide burn deaths^6–8^. Therefore, more studies are needed to investigate the epidemiology, etiology and outcomes of burn populations in this region in order to further improve the effects of preventive measures to mortality and deformities. As Peck et al. mentioned^9^, a detailed understanding of epidemiology is essential in order to take steps to prevent injuries. However, most of the previous studies on burn epidemiology and outcomes are limited to vulnerable age groups such as children^10–14^ and elderly population or a specific type of burn such as thermal burns or chemical burns^15,16^. The adults between the age of 18 and 60 years old have not been fully studied although they occupied the largest number of burn patients. Besides, their post-burn recovery and rehabilitation becomes especially important considering the primary responsibilities they have taken to society and family. Most of them are backbone of the family regarding physical, emotional and economic support. This retrospective study has therefore been performed to investigate the burn epidemiology and outcomes in the southwest of China over a long-time span of 17 years (2002–2019), which in particular analyzed the epidemiology and outcomes among young and middle-aged adults between 18 and 40 years old.

## Materials and methods

### Study setting

The data were collected in the Institute of Burn Research (IBR), located in the first affiliated with the Army Medical University (AMU). This burn specialized center is one of the earliest department for burns in China and is also one of the largest burn centers across the world with 150 inpatient beds (including 18 ICU beds). An estimated of 1300 burn patients are admitted to the center annually. The year of 2018 was the 60th anniversary of this center; and a lot of achievements on burn treatment has been reached including the Chinese Rule of Nine, fluid resuscitation protocol, experience in inhalation injury, wound treatment strategies, prevention and treatment of burn infections^17^.

Another significant achievement of this center was to develop the first and the biggest burn database in the mainland of China. This database could directly access to the hospital information system (HIS), the laboratory information management system (LIS) and the electronic medical record (EMR). This database was established to provide a comprehensive and convenient access to the information of burn inpatients and outpatients to clinical professionals and researchers (with access permission) for the purpose of case review and therefore promoting clinical practice, research and burn prevention.

### Data extraction

We reviewed all 18,138 cases with the diagnosis of burn admitted to the Institute of Burn Research between January 2002 and December 2019. Fifty-one duplicates were removed according to name, gender and birth date; and another 148 records were excluded due to data missing. Eventually, a total of 17,939 cases were included for final analysis. Demographic and clinical information of burn patients were collected including age, gender, burn size and depth, burn sites, inhalation injury and the outcomes (including length of stay, length of stay in BICU, total cost and count of death).

All methods were performed in accordance the Declaration of Helsinki. This study was a retrospective analysis, and no individual information that may identify the patients were not reported; therefore, informed consent from the patients were not required. The need for informed consent was waived and the study protocol was approved by the medical ethics committee in southwest hospital affiliated to the Third Military Medical University (Army Medical University) (approval number: KY201904).

### Statistical analysis

The data were primarily entered and processed using Microsoft Excel 2010 (Microsoft Corporation). The data analysis was performed and the figures were drawn using the GraphPad Prism 5 (GraphPad Software Inc, San Diego, CA). The software of Statistical Package for the Social Sciences 21 (SPSS Inc, Chicago, IL) was adopted to analyse the descriptive statistics and headcounts. Pearson chi-square test or Fisher’s exact test was used to compare the patient numbers in different groups. T-tests or one-way ANOVA were used to compare two or more quantitative variables (e.g., LOS, LOS in ICU, cost, ABSI and BI score), and Scheffe’s test was performed as a post-hoc test in the comparison of two groups. The Abbreviated Burn Severity Index (ABSI)^12^ and the index of burn severity (Burn Index, BI) were calculated as follows: ABSI = Gender (female = 1, male = 0) + Age (0–20 = 1, 21–40 = 2, 41–60 = 3, 61–80 = 4, 80–100 = 5) + Inhalation injury (yes = 1, no = 0) + Full-thickness burns (yes = 1, no = 0) + Total body surface area (TBSA) (1–10% = 1, 11–20% = 2, 21–30% = 3, 31–0% = 4, 41–50% = 5, 51–60% = 6, 61–70% = 7, 71–80% = 8, 81–90% = 9, 91–100% = 10); BI = TBSA of the full-thickness burn% + 1/2 TBSA of the deep partial-thickness burn%^12^.

Multiple linear regression of approach (entry: *P* = 0.05; removal: *P* = 0.10) was used to examine the factors that interpreting the medical cost. Multiple logistic regression (entry: *P* = 0.05; removal: *P* = 0.10) was used to screen the factors contributing to mortality. *P* values < 0.05 were considered significant.

### Ethics approval and consent to participate

Our study has received ethics approval from southwest hospital affiliated to the Third Military Medical Universitiy (Army Medical University) (approval number: KY201904).

## Results

### Demographic characteristics

**Figure 1:**
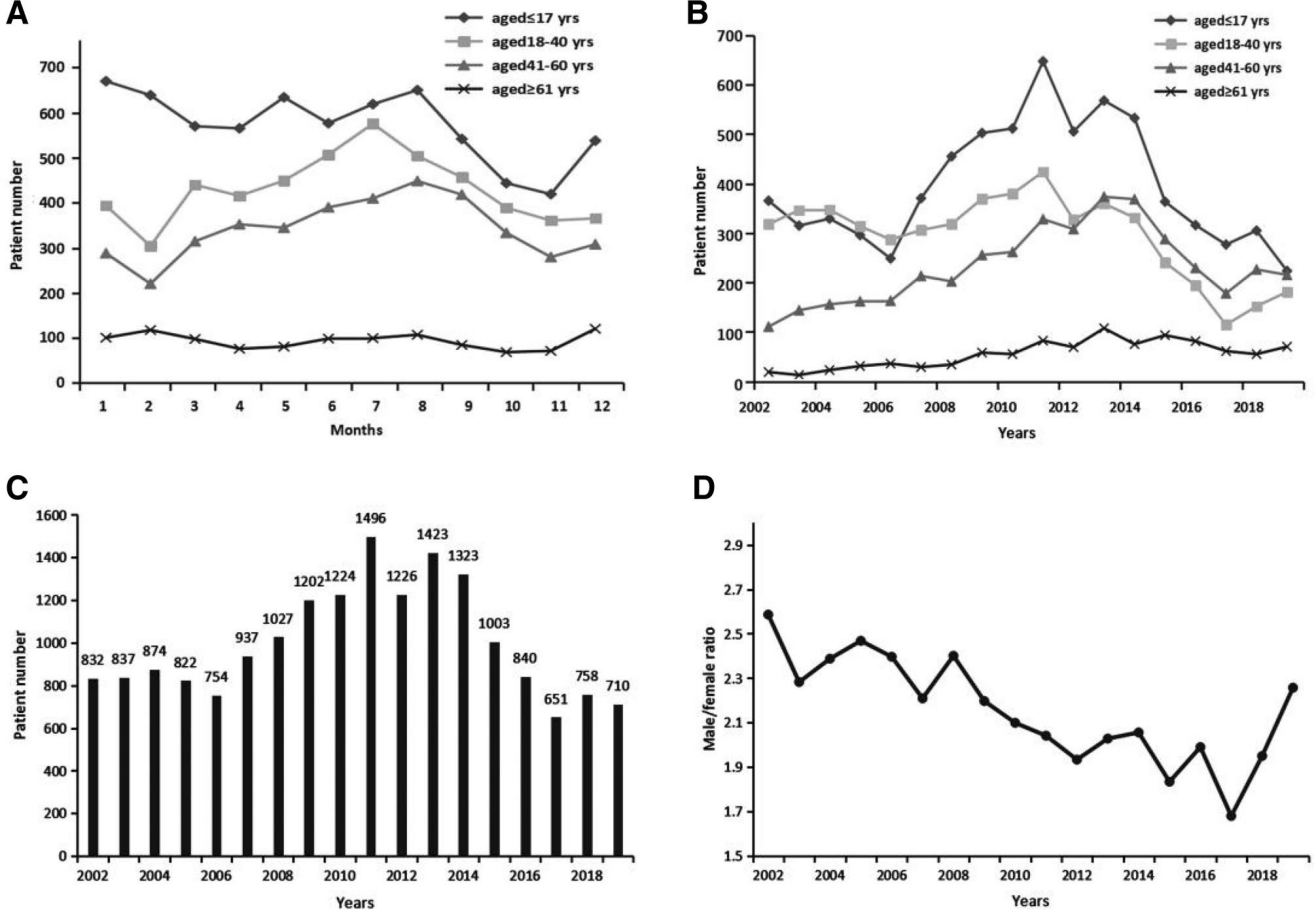
Patient demographic distribution. (**A**) Distribution of different age groups of patients by months. (**B**) Distribution of different age groups of patients by years. (**C**) Distribution of the number of the total patients by years. (**D**) The distribution of the ratio of male to female by years.

Table 1 and Figure 1 illustrated the general characteristics of the burn patients investigated in this study. The age of the 17,939 burn patients ranged from 257 days to 95 years old. The patients under the age of 18 years old accounted for the highest proportion of patients with a total number of 7192 (40.1%) and followed by the number of young and middle-aged adults between 18 and 40 years old accounting for nearly a third of all the patients (5383, 30.0%).

**Table 1:** Distribution of demographics and clinical features by age and gender.

Item	Age				*P* Value	Gender		*P* Value
	≤ 17 (n = 7192)	18–40 (n = 5383)	41–60 (n = 4265)	≥ 61 (n = 1099)		Male (n = 12204)	Female (5735)	
**Gender (%)**								
Male	4340 (60.3)	4105 (76.3)	3129 (73.4)	630 (57.3)	< 0.001	NA	NA	NA
Female	2852 (39.7)	1278 (23.7)	1136 (26.6)	469 (42.7)		NA	NA	NA
**Etiology (%)**								
Scald	5393 (75.0)	1448 (26.9)	1363 (32.0)	401 (36.5)	< 0.001	5068 (41.5)	3537 (61.7)	< 0.001
Flame	1531 (21.3)	3334 (61.9)	2417 (56.7)	610 (55.5)		5920 (48.5)	1972 (34.4)	
Electrical	216 (3.0)	533 (9.9)	417 (9.8)	48 (4.4)		1080 (8.8)	134 (2.3)	
Chemical	4 (0.1)	13 (0.2)	10 (0.2)	3 (0.3)		23 (0.2)	7 (0.1)	
Other	48 (0.7)	55 (1.0)	58 (1.4)	37 (3.4)		113 (0.9)	85 (1.5)	
**Head/face/neck (%)**								
No	4307 (59.9)	2632 (48.9)	2262 (53.0)	749 (68.2)	< 0.001	6440 (52.8)	3510 (61.2)	< 0.001
Yes	2885 (40.1)	2751 (51.1)	2003 (47.0)	350 (31.8)		5764 (47.2)	2225 (38.8)	
**Trunk (%)**								
No	3604 (50.1)	3361 (62.4)	2512 (58.9)	691 (62.9)	< 0.001	7005 (57.4)	3163 (55.2)	0.005
Yes	3588 (49.9)	2022 (37.6)	1753 (41.1)	408 (37.1)		5199 (42.6)	2572 (44.8)	
**Limbs (%)**								
No	2422 (33.7)	1674 (31.1)	1200 (28.1)	319 (29.0)	< 0.001	3926 (32.2)	1689 (29.5)	< 0.001
Yes	4770 (66.3)	3709 (68.9)	3065 (71.9)	780 (71.0)		8278 (67.8)	4046 (70.5)	
**Hands (%)**								
No	6441 (89.6)	4331 (80.5)	3553 (83.3)	962 (87.5)	< 0.001	10217 (83.7)	5070 (88.4)	< 0.001
Yes	751 (10.4)	1052 (19.5)	712 (16.7)	137 (12.5)		1987 (16.3)	665 (11.6)	
**Feet (%)**								
No	6946 (96.6)	5056 (93.9)	3914 (91.8)	973 (88.5)	< 0.001	11443 (93.8)	5446 (95.0)	0.002
Yes	246 (3.4)	327 (6.1)	351 (8.2)	126 (11.5)		761 (6.2)	289 (5.0)	
**Perineum (%)**								
No	6477 (90.1)	5200 (96.6)	4090 (95.9)	1045 (95.1)	< 0.001	11414 (93.5)	5398 (94.1)	0.133
Yes	715 (9.9)	183 (3.4)	175 (4.1)	54 (4.9)		790 (6.5)	337 (5.9)	
**Hip (%)**								
No	5862 (81.5)	4972 (92.4)	3829 (89.8)	948 (86.3)	< 0.001	10739 (88.0)	4872 (85.0)	< 0.001
Yes	1330 (18.5)	411 (7.6)	436 (10.2)	151 (13.7)		1465 (12.0)	863 (15.0)

### Distribution of burns

#### Seasonality

Figure 1A showed a change of burn incidence over month in different age groups. For the elderly patients over the age of 61 years old, the incidence of burns kept at an average level in every month. For adult patients under the age of 60 years old, the burns that most frequently occurred were during summer season from June to September while the occurrence stayed in a low level during autumn season from October to December; and the number increased to the peak in July and reduced to the nadir in February. For pediatric patients, burns occurred most frequently during Chinese spring festival of January and February while in November it showed the lowest occurrence of burns.

#### Year

A similar change trend was indicated in the Fig. 1B for the age groups of pediatric patients and adult patients under the age of 60 years old, increasing first to the peak in 2011 and then kept dropping year by year. However, for the elderly burn patients, the number has kept the trend of increase as the year went on. As the Fig. 1C shown, the total number of burn patients kept increasing year by year until the year of 2013 and reached at a high level in the year of 2011 (n = 1496), 2012 (n = 1226), 2013 (n = 1423), and 2014 (n = 1323). Since then, the number began to drop, and the decrease kept going on, and reached at the nadir in 2017 (n = 651).

#### Gender

The ratios of males to females presented a significant decline across the seventeen years and kept dropping from 2.59:1 in 2002 to 1.68:1 in 2017, with a slight increase after that in 2018 and 2019 (Fig. 1D). Moreover, among the adult patients between the age of 18 and 40 years old, the number of male patients was far larger than that of female patients with an estimated ratio of 3.22:1 (Table 1).

#### Age, gender and season difference on burn causes

Scalding was the leading cause of burns, accounting for nearly a half of all cases (48.0%), and the second most common cause was flame, accounting for 44.0% of all patients. Electrical, chemical, and other types of burns accounted for 6.8%, 0.2%, and 1.1% of all the patients, respectively (Table 1).

**Figure 2:**
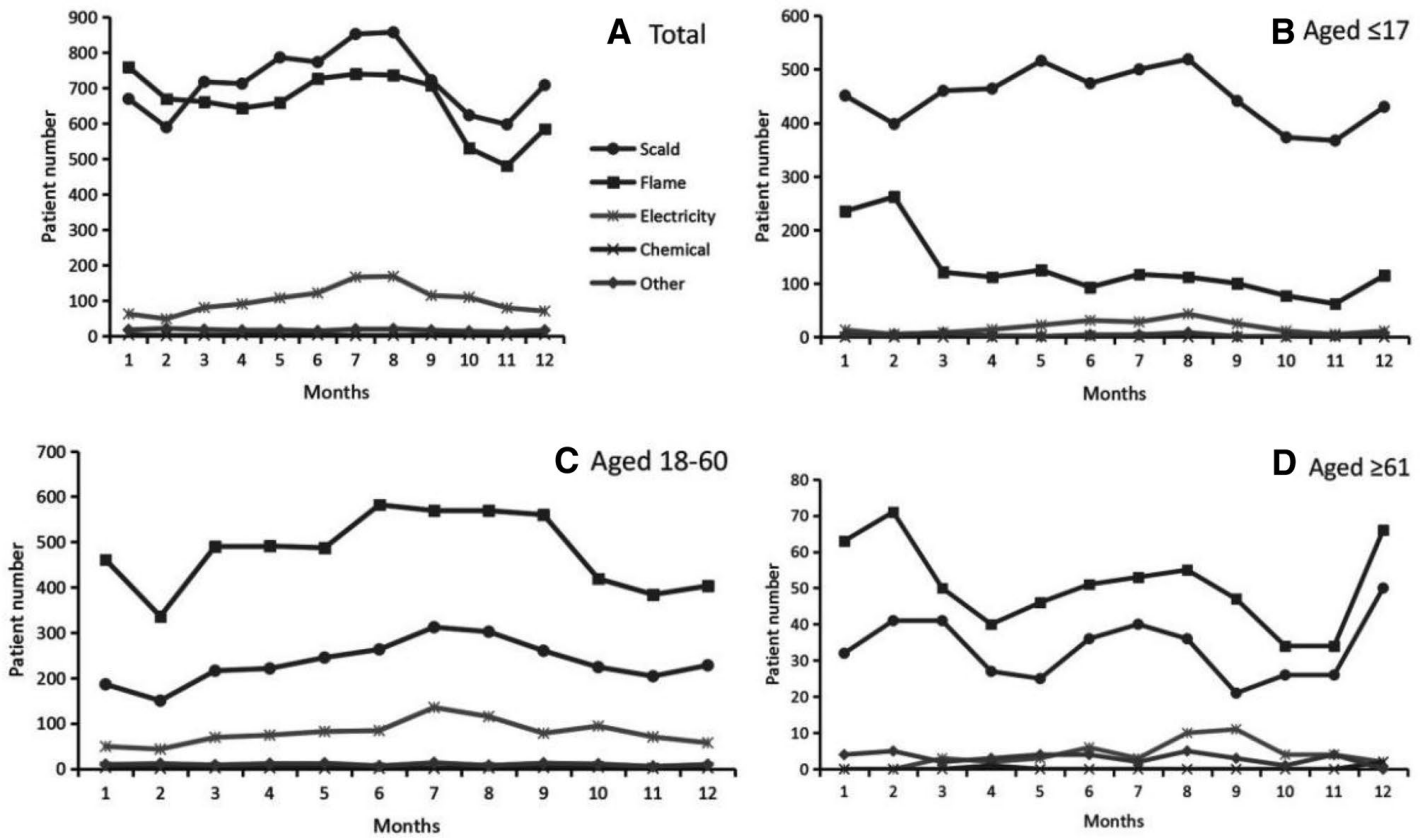
Distribution of burn causes by months in different age groups. (**A**) In total patients. (**B**) In patients under the age of 18 years old. (**C**) In patients aged between 18 and 60 years old. (**D**) In patients above the age of 60 years old.

**Figure 3:**
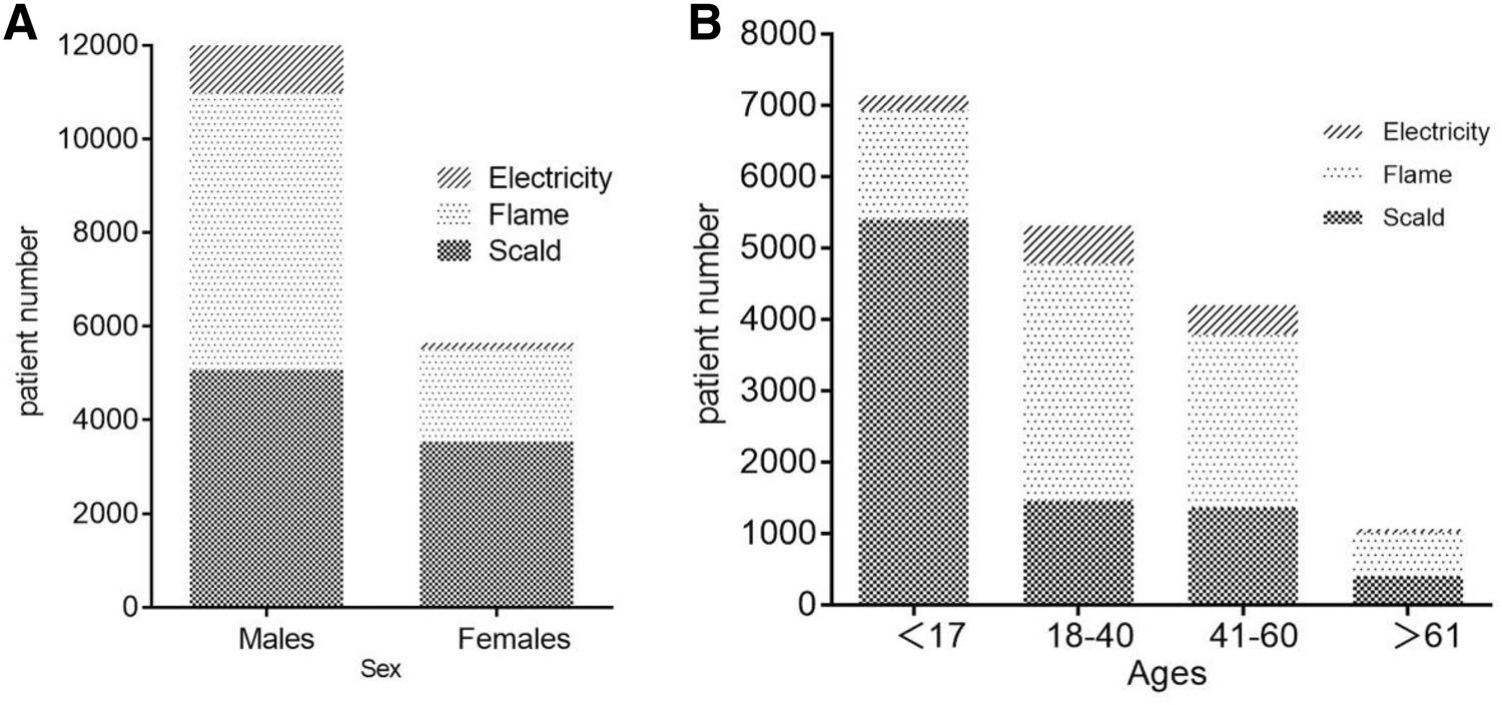
Etiology analysis. (**A**) Distribution of etiology by ages. (**B**) Distribution of etiology by gender.

Scalding and flame were indicated as the two most common causes of burns in all groups (Fig. 2A). However, the leading cause of burns was significantly different in pediatric patients and adult patients. The cause of scalding was in the first place among pediatric patients, with the number accounting for almost three fourth of all the patients (5393, 75.0%) (Figs. 2B, 3B); In comparison, flame was the most common cause of burns in adult patients under the age of 60 years old (5751, 59.61%) (Figs. 2C, 3B).

It was also showed the gender difference on the causes of burns. The incidence of burns caused by flames was significantly larger than the burns caused by scalding among male patients (48.24% versus 41.08%, *P* < 0.001); while the burn caused by scalding was significantly larger than that caused by flames among female patients (61.04% versus 33.41%, *P* < 0.001) (Fig. 3A).

As shown in the Fig. 2B, flame burns in pediatric patients occurred most frequently in January and February. The incidence of burns caused by scalding stayed in a high level across spring and summer seasons from March to August compared to a low level in the autumn season from October to November. Different from the pediatric patients, the number of burns caused by flame and scalding in adult patients was both reaching at the nadir in February (Fig. 2C). And the number of scalding burns reached at peak in July while the flame burns stayed in a high level across spring and summer seasons from March to September. Like the pediatric patients, the incidence of flame burns in the elderly patients aged over 60 years old reached at the peak in February and reached at the nadir in October (Fig. 2D). Regarding the scalding burns, there were three periods with high level of incidence: from February to March, from June to August, and the month of December.

### Burn sites

As shown in Table 1, Limbs, head/face/neck, and trunk were the most frequently occurred burn sites, with the number and the percent of 12,324 (68.7%), 7989 (44.5%), and 7771 (43.3%), respectively. The top three burn sites in all adult patients aged 18 years old and above were in sequence of limbs (7554, 70.3%), head/face/neck (5104, 47.5%) and truck (4183, 38.9%); the top three burn sites in pediatric patients were in sequence of limbs (4770, 66.3%), trunk (3588, 49.9%) and head/face/neck (2885, 40.1%).

### Burn severity and cure rates

The average total body surface area (TBSA) was 13.64 ± 16.83% (median 8%) with a range of 0.1–100%. Patients with TBSA of 0–10% and 11–20% accounted for 11296 (62.97%) and 3496 (19.49%) of all cases, respectively. A total of 874 (4.87%) patients had TBSA > 50%. With the improvement of burn treatment, the cure rates of burn patients have continuously increased in the last 17 years. As shown in Table 2, the results indicated great improvements in treatment outcomes in our burn center. The cure rates among patients with ≤ 90% TBSA remained a high level from 78.30% to 99.59%. For severe burns with ≥ 91% TBSA, the cure rates have dramatically increased from 23.08% in 2002–2005 to 76.47% in 2016–2020.

**Table 2:** The distribution of cure rates by burn severity.

Cure rates	Burn severity [n, (%)]			
	≤ 50% TBSA	51–70% TBSA	71–90% TBSA	≥ 91% TBSA
2002–2005	3122 (99.5)	79 (91.9)	48 (80.0)	6 (23.1)
2006–2010	4828 (99.6)	103 (93.6)	72 (80.9)	19 (54.3)
2011–2015	6152 (99.1)	159 (94.1)	83 (78.3)	19 (51.4)
2016–2020	2815 (98.0)	82 (89.1)	40 (85.1)	13 (76.5)

Table 3 presented the burn severity of ABSI index in different age groups. The results suggested that the elderly patients were most likely to suffer severe burns compared to other age groups. The majority of the pediatric patients (4890, 68.0%) had a very low ABSI burn score; while the majority of the adult patients under the age of 60 years old had a moderate to severe ABSI burn score (7576, 78.5%). The elderly patients had a moderately severe to serious ABSI burn severity.

**Table 3:** Distribution of burn severity by age.

ABSI burn score (score scale)	Age [n (%)]				Gender [n (%)]	
	≤ 17 (n = 7192)	18–40 (n = 5383)	41–60 (n = 4265)	≥ 61 (n = 1099)	Male (n = 12204)	Female (n = 5735)
Very low (2–3)	4890 (68.0)	1723 (32.0)	0	0	5281 (43.3)	1332 (23.2)
Moderate (4–5)	1886 (26.2)	2728 (50.7)	2586 (60.6)	137 (12.5)	4630 (37.9)	2707 (47.2)
Moderately severe (5–6)	305 (4.2)	444 (8.2)	1006 (23.6)	715 (65.1)	1255 (10.3)	1215 (21.2)
Serious (6–7)	75 (1.0)	200 (3.7)	298 (7.0)	160 (14.6)	459 (3.8)	274 (4.8)
Severe (8–9)	21 (0.3)	141 (2.6)	173 (4.1)	54 (4.9)	279 (2.3)	110 (1.9)
Maximum (≥ 10)	15 (0.2)	147 (2.7)	202 (4.7)	33 (3.0)	300 (2.5)	97 (1.7)

### Health outcomes

The presence of a burn with an inhalation injury was confirmed in 543 patients (3.02%) (Table 4), with the lowest occurrence rate in pediatric patients (0.8%). The average LOS was 32.11 ± 65.72 days (median: 17 days). The adult patients aged between 18 and 60 years old confirmed the longest length of stay in hospital with more than 40 days. The length of staying in BICU was significantly higher in burn patients between the age of 40 and 60 years old. A significantly longer stay in hospitals and BICU were revealed among male patients compared to female ones, with an average of 9 days longer in hospital and 0.5 day longer in BICU. Patients injured by flame and electrical burns spent significantly longer time in hospitals and BICU, compared to those patients caused by scald, chemical and other reasons, with an average of more than 20 days longer.

**Table 4:** Distribution of burn severity and burn outcomes by age, gender and etiology.

	With inhalation injury N (%)	Length of stay (LOS) Mean (SD)	Length of stay in ICU Mean (SD)	Cost Mean (SD)	ABSI Mean (SD)	BI Mean (SD)
**Age (years)**						
≤ 17	55 (0.8)	19.20 (24.7)	0.43 (3.5)	18176.31 (46418.7)	3.27 (1.3)	6.98 (8.3)
18–40	201 (3.7)	43.22 (92.4)	1.51 (10.2)	68136.20 (182373.6)	4.58 (2.2)	9.58 (15.2)
41–60	237 (5.6)	42.10 (74.6)	2.38 (11.5)	93133.29 (222298.8)	5.93 (2.3)	10.92 (16.4)
≥ 61	50 (4.5)	23.47 (31.1)	1.62 (8.9)	45121.61 (93454.1)	6.90 (1.7)	8.19 (12.2)
*P* value	< 0.001	< 0.001	< 0.001	< 0.001	< 0.001	< 0.001
**Gender**						
Males	421 (3.4)	35.68 (75.3)	1.47 (9.3)	61631.94 (174901.2)	4.31 (2.3)	9.24 (13.9)
Females	122 (2.1)	24.52 (36.7)	0.91 (6.8)	33504.29 (98215.4)	4.95 (2.0)	7.77 (11.2)
*P* value	< 0.001	< 0.001	< 0.001	< 0.001	< 0.001	< 0.001
**Etiology**						
Scald	40 (0.5)	20.85 (31.0)	0.45 (3.9)	24468.55 (81920.0)	3.91 (1.8)	6.65 (9.2)
Flame	497 (6.3)	40.41 (85.2)	2.19 (11.6)	78666.34 (203600.8)	5.17 (2.6)	11.83 (16.5)
Electrical	6 (0.5)	58.70 (87.1)	1.61 (9.7)	87808.60 (164215.0)	4.48 (1.3)	5.08 (8.4)
Chemical	0 (0.0)	25.67 (20.2)	0.50 (2.4)	33719.24 (45986.7)	4.73 (1.5)	5.64 (7.6)
Other	0 (0.0)	28.56 (28.3)	0.14 (1.7)	26795.12 (35891.0)	4.73 (1.3)	2.20 (2.7)
*P* value	< 0.001	< 0.001	< 0.001	< 0.001	< 0.001	< 0.001

Table 5 showed the multiple linear regression of factors contributed to the total medical cost. The demographic information of age and gender, the cause of burns, BI score, the thickness of burns, and the length of stay in hospital were included as contributors. Compared to the reference item (Ref), the higher of the absolute value of the β score, the higher contribution of the item to the medical cost. The results suggested that electrical burns, the gender of male, and the middle age between 41 and 60 years old showed significant association with higher medical cost.

**Table 5:** Multiple linear regressions of factors to the total medical cost.

	β (95% CI)	SD	P
**Constant**	− 27998.0 (− 31996.7, − 23999.3)	2040.1	< 0.001
**BI**	4123.1 (3987.6, 4258.6)	69.1	< 0.001
**Length of stay in burn institute**	712.9 (687.5, 738.3)	13.0	< 0.001
**Length of stay in ICU**	5864.6 (5674.1, 6055.1)	97.2	< 0.001
**Full-thickness burns**	9823.2 (6269.1, 13377.4)	1813.3	< 0.001
**Year of admission**	4678.2 (4354.6, 5001.9)	165.1	< 0.001
**Age**			
≤ 17	Ref	Ref	Ref
18–40	11862.1 (7818.6, 15905.6)	2062.9	< 0.001
41–60	26798.1 (22568.0, 31028.3)	2158.1	< 0.001
≥ 61	8756.1 (1972.1, 15540.1)	3461.1	< 0.05
**Gender**			
Male	Ref	Ref	Ref
Female	− 6619.0 (− 9953.1, − 3284.9)	1701.0	< 0.001
**Etiology**			
Flame	Ref	Ref	Ref
Scald	992.8 (− 2573.4, 4559.0)	1819.4	0.59
Electrical	20719.9 (14065.0, 27374.9)	3395.2	< 0.001
Chemical	− 2738.5 (− 39812.8, 34335.7)	18914.5	0.89
Others	6514.4 (− 8239.5, 21268.4)	7527.2	0.39

β means nonstandard coefficients. Ref means reference item, all other items showed the relative difference value compared to the reference item. The higher of the absolute value of the β score, the higher of the contribution to the medical cost is.

In total, there were 145 deaths among the 17939 patients, for a mortality of 0.81%. Our results showed in Table 6 that older age with more than 41 years old had the greatest influence on mortality (OR 3.38, *P* < 0.001), followed by full-thickness burns (OR = 3.09, *P* < 0.05) and older age with more than 61 years old (OR 2.88, *P* < 0.05). Moreover, a shorter length of stay in hospitals (OR 0.97, *P* < 0.001) and female gender (OR 0.57, *P* < 0.05) were protective factors for mortality.

**Table 6:** Logistic regression of factors related to the mortality.

	β	SE	OR	95% CI	P
**BI**	0.07	0.00	1.08	(1.07, 1.09)	< 0.001
**Full-thickness burns**	0.68	0.34	1.98	(1.02, 3.87)	< 0.05
**Year of admission**	− 0.15	0.02	0.86	(0.83, 0.90)	< 0.001
**Age**					
≤ 17	Ref	Ref	Ref	Ref	Ref
18–40	0.17	0.34	1.19	(0.61, 2.32)	0.61
41–60	0.83	0.33	2.30	(1.20, 4.42)	< 0.05
≥ 61	1.23	0.48	3.41	(1.34, 8.69)	< 0.05
**Gender**					
Male	Ref	Ref	Ref	Ref	Ref
Female	− 0.34	0.27	0.71	(0.43, 1.18)	0.18

### Framework of severe burn management continuum

**Figure 4:**
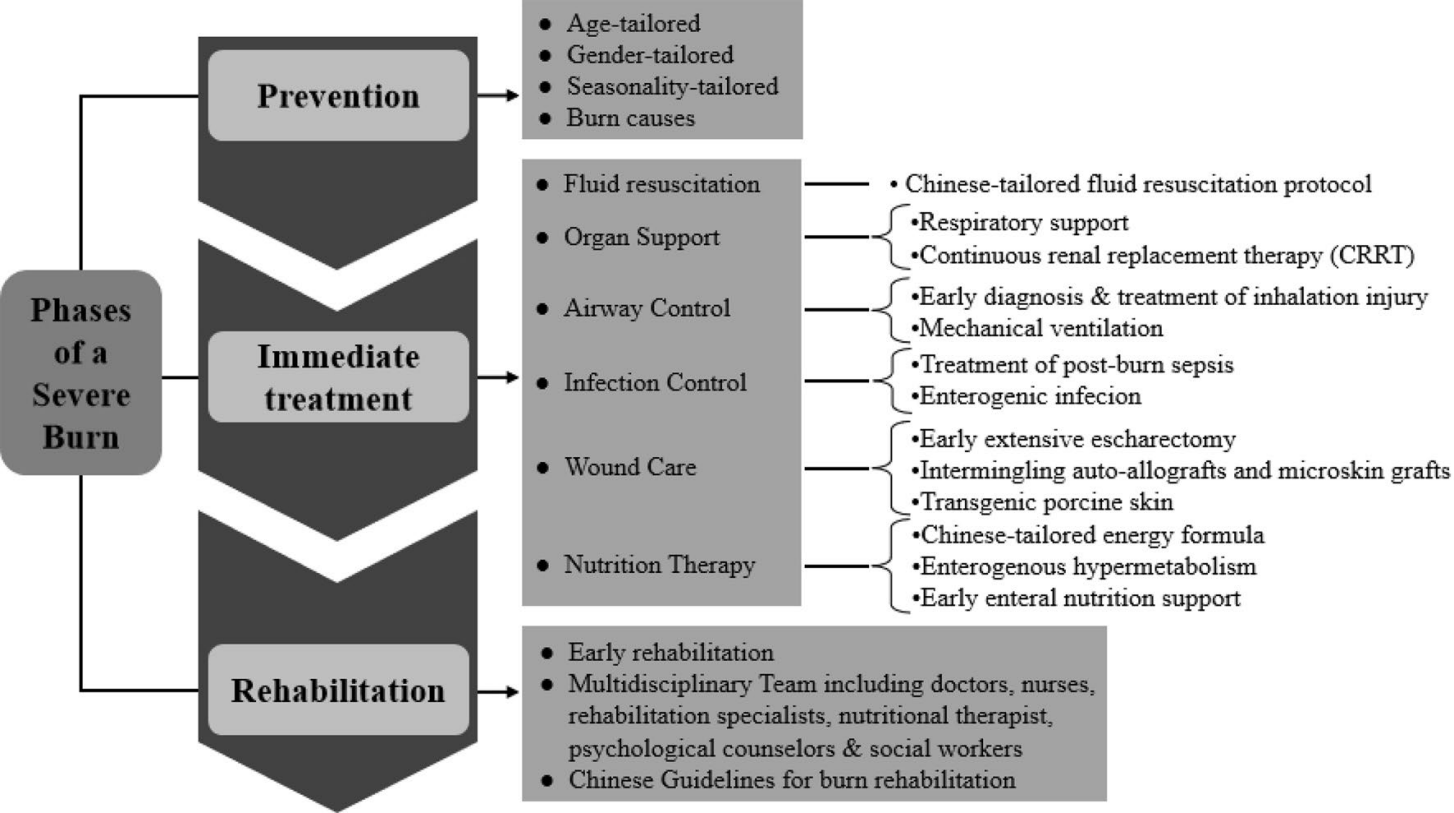
Burn prevention and management continuum.

Figure 4 described the model of burn management continuum used for developing strategies to prevent and manage severe burns. This model was designed to decrease burn injuries to populations, and promote recovery and rehabilitation to burn patients. It was worthy to note that this model presented a continuous process of burn management from the stage of burn prevention to burn treatment and finally to burn rehabilitation and reconstruction.

Prevention was the process designed to prevent or minimize the risks related to burn injuries. As our data showed, with the improvement of burn prevention strategies, the number of burn injuries has continuously declined since the year of 2011 (Fig. 1C). Notably, the effect of gender, age and seasonality on occurrence of burn injuries was significant. Another factor should be considered was the top two causes of burn injuries: scalding and flame. All these results suggested a prevention strategy should be tailored according to the population’s characteristics. For example, the public education should be different between teenagers and the elderly.

The phase of in-hospital treatment encompassed the immediate actions taken in the face of a severe burn. The six aspects and related strategies in the framework were developed on the basis of our accumulated and extensive experience through treatment of large number of cases. Most importantly, these aspects have drawn from clinical and basic research and from the experience of translating the basic findings into clinical practice. Some strategies such as estimation of burn area, fluid resuscitation protocol and energy formula were developed and tailored according to the characteristics among Chinese populations.

The phase of rehabilitation was important for severe burn patients to recover from the impact of burns and return to society. Once immediate needs were met, the rehabilitation phase could begin. Our institute was one of the pioneers of implementing early rehabilitation for burn patients in Mainland China. The burn rehabilitation have gained increase attention since 1995 in our center, and a whole floor of special burn rehabilitation center was established in 2011, comprising of multidisciplinary team including doctors, nurses, rehabilitation specialists, nutritional therapist, psychological counselors & social workers.

## Discussion

Burn patients accounted for a large proportion of hospitalized injury patients worldwide. They suffered from great physical and psychological burden because of the associated morbidity, rehabilitation, mortality and requirement of high cost medical services. Therefore, investigations on epidemiological parameters related to burns and treatment outcomes could assist with the adoption of effective interventions and individualized prevention approaches in southwest China. In particular, this study focused on the clinical characteristics and treatment outcomes of different age groups of burn patients. The primary result we found was that the annual number of burn injuries has kept decreasing since the year of 2014, which was consistent with the global trends^18,19^. This change might be partially attributed to the increased awareness and education of burn prevention and the improved burn-preventative environment. Another notable finding was that the gender difference and age difference should be considered when making individualized interventions and rehabilitative treatments. The etiology, the frequently burn sites, the occurrence rate of inhalation injury, the LOS, the cost and the mortality rates were significantly different between male patients and female patients, and among patients of different age groups. However, the burn severity showed by the indicators of TBSA, ABSI and BI, and the economic burden indicated by the medical cost were still in a high level, which suggested that the current prevention and care of burns remained inadequate, demonstrating that more effective interventions should be introduced in the future.

Same as the results from other studies^20–22^, scalding and flame were identified as the two most common causes of burning in the southwest of China. Furthermore, the age difference and gender difference on etiology should be noticed. Firstly, considering the age difference, the scalding was in the first cause to burn injuries among pediatric patients, comprising of 74.24% of all cases under the age of 18 years old. Scald burns were primarily caused by hot steam, hot water, hot soup and hot oil. This suggested that hot steam and hot fluid should be cautioned among children population. In contrast, flame burns were the leading etiology in adult patients. The patients between the age of 18 and 40 years suffered most from flame burning, accounting for 62.07% of all the cases in this age group. Flame injuries were mainly generated by gas and bomb explosion, short circuit of electricity and fireworks. Secondly, considering the gender difference, the incidence of burns caused by flames was significantly larger than those caused by scalding among male patients; while the burns caused by scalding was significantly larger than those caused by flames among female patients. Electricity was the third most common cause of burns in the pediatric population and the adult patients under the age of 60 years old; while chemical burns was the third cause in the elderly population above the age of 60 years old. It was worthwhile to note that although the flame and scald were the first two etiologies of burn injuries, the electrical burn was the one that costed the highest medical expense (Table 5). According to the previous studies, this was likely because that 10–68%^23^ of the electrical burns resulted in amputation, which increased the medical cost. The above findings indicated that burn education was necessary for the population of all ages, and particularly preventive strategies should be individualized by age, gender, and burn causes. For example, children were curious about their surroundings but they were too young to be aware of burn-related dangers. Therefore, it was their parents or guardians as the main target of education about providing safe environment and eliminating possible burn dangers to their children, such as putting the hot water or oil away from children and protecting the electrical plugs in case of electricity burns.

Our findings demonstrated that the adult patients aged between 18 and 60 years old were the main victims of burn injuries, accounting for 53.8% of the total cases. The first cause to burns in this population was the flame, followed by scald, and then the electricity. This may relate to the potential burn-related hazards in work place^16^. In particular, the number of male patients at this age was significantly larger than that of female patients, with the ratio of three to one (Table 1). Moreover, almost half of those patients were self-funded on the medical expense; and the male patients spent significantly more money on treatment compared to the cost among female patients. Furthermore, the sites of head/face/neck, trunk and limbs were the most significantly parts of body that burn occurred. During the process of post-burn recovery and rehabilitation, the scar was a notable problem. The scar on head/face/neck will influence the facial appearance, and the scar on limbs will affect the physical functions. All of these results suggested that the adult patients especially the male patients should become the major prevention target in the future considering their responsibilities to the family and society. Additionally, the early rehabilitation on physical abilities should be emphasized.

When great awareness and focus has been placed on the elderly and young children, the population of females should be also considered as a vulnerable group of burn injuries and worthy of increased attention. It was interesting to find out that the percentage of female burn patients increased annually despite of the decreased number of the overall adult patients (Fig. 1D). It suggested that the risk for Chinese females to suffer from burn injuries kept increasing. After further analysis of the etiology, we found that scalding was the priority cause to burns in females, accounting for over 60 percent of the total female patients (Table 1). Surprisingly, in another developing country India, similar finding has been reported before that Indian population of young females between the ages of 16 and 35 years were high-risk of burning. The reason lied in that females cooked over open flames at floor level, often with faulty equipment and loose clothing susceptible to catching fire. Based on years of clinical experience on burn center, our research team has discussed the reasons for the high risk of burning in Chinese females. Considering the priority etiology of scalding, we assumed the first reason was that women still took the main responsibility of cooking for the family, so they were more likely exposure to hot water/oil and cooking flames compared to men. Secondly, more and more women were involved in the workforce. The results showed that the labor force participation rate in Chinese females reached 70%^24^. And the chance of exposure to burn risks was increasing if the precautions were not sufficient in the workplace. The third reason we considered may be due to the culture factor of gender inequality in developing countries. Women in those countries such as India and China were mainly work in the areas with relatively high risk of burn injuries such as housemaid and catering services.

Building on the previous findings^19^ of those the burn size determining major complications and survival rates, this study also identified that the burn size and depth contributed to the total cost of burn patients according to burn index (BI). BI was an indicator of burn severity calculated on the basis of TBSA and burn depth. In Dr. Jeschke and Prof. Herndon’s study of an sample of 952 severely burned pediatric patients, they confirmed that burn size of 62% TBSA was a crucial threshold for post-burn morbidity and mortality^25^; and the cost of burn treatment was increasing accordingly. Besides the TBSA, ABSI, and BI, there were other indicators developed to predict burn outcomes such as the modified Baux score^26^ and the Pediatric Risk of Mortality (PRISM) score^27^. Most of these indices were formula developed by calculating risk factors for post-burn morbidity and mortality. For example, the Baux score was calculated in consideration of age, burn size and the presence of inhalation injury. Further analysis and comparisons were needed to figure out which of the above indicators would be more accurate and effective in predicting mortality and hospital length of stay among patients with burns.

Some limitations should be noted when interpreting these findings. First, due to system default, we could not access to the data of other poor outcomes such as infection and sepsis. Therefore, we failed to find out the risk factors contributed to these poor outcomes. However, our research team is now conducting a longitudinal study trying to investigate risk factors for these poor outcomes in Chinese burn patients. Another limitation was that our data only partially reflected the epidemiology of burn injuries in southwest of China, and the patients in this study mainly originated from Chongqing, Sichuan, Yunnan and Guizhou Province. Due to unequal economic development between the south cities and the east cities in China, the findings cannot represent the status in the eastern cities of China. Therefore, more studies with large sample sizes and multiple centers are still needed. Thirdly, since our center is in a tertiary hospital, so some of the severe burn patients have received treatments from other hospitals before they were transferred into our center. Therefore, the burn severity observed in our study might be higher than average.

## Conclusion

This 17-year retrospective study examined the epidemiology and outcomes of burn patients in southwest of China. The annual number of burn injuries has kept decreasing, which was partially attributed to the increased awareness and education of burn prevention and the improved burn-preventative circumstances. However, the burn severity and the economic burden were still in a high level. And the gender difference and age difference should be considered when making individualized interventions and rehabilitative treatments.

## Data Availability

The datasets used and/or analysed during the current study are available from the corresponding author on reasonable request.

## Abbreviations

AMU: Army Medical University
IBR: Institute of Burn Research
HIS: Hospital Information System
LIS: Laboratory Information Management System
EMR: Electronic Medical Record
ABSI: Abbreviated Burn Severity Index
BI: Burn Index
TBSA: Total Body Surface Area
LOS: Length of Stay
